# Pathophysiological Significance of Neutrophilic Transfer RNA-Derived Small RNAs in Asymptomatic Moyamoya Disease

**DOI:** 10.3390/cells10051086

**Published:** 2021-05-01

**Authors:** Lingzhi Li, Ping Liu, Rongliang Wang, Yuyou Huang, Jichang Luo, Liqun Jiao, Zhen Tao, Yangmin Zheng, Junfen Fan, Haiping Zhao, Ziping Han, Yumin Luo

**Affiliations:** 1 Department of Neurology, Institute of Cerebrovascular Diseases Research, Xuanwu Hospital, Capital Medical University, Beijing 100000, China; lingzhi08@ccmu.edu.cn (L.L.); pingliu@xwhosp.org (P.L.); wangrongliang@xwhosp.org (R.W.); yuyouhuang@ccmu.edu.cn (Y.H.); taozhen@xwhosp.org (Z.T.); zhengyangmin@xwhosp.org (Y.Z.); fanjunfen@xwhosp.org (J.F.); zhaohaiping@xwhosp.org (H.Z.); 2 Beijing Key Laboratory of Translational Medicine for Cerebrovascular Diseases, Beijing 100000, China; 3 Department of Neurosurgery, Xuanwu Hospital, Capital Medical University, Beijing 100000, China; luojichang@mail.ccmu.edu.cn (J.L.); jiaoliqun@xwhosp.org (L.J.); 4 Beijing Institute for Brain Disorders, Beijing 100000, China

**Keywords:** asymptomatic moyamoya disease, inflammation, neutrophil, next-generation RNA sequencing, transfer RNA-derived small RNA

## Abstract

Understanding asymptomatic moyamoya disease (aMMD), for which treatment options are currently limited, is key to the development of therapeutic strategies that will slow down the progression of this disease, as well as facilitate the discovery of therapeutic targets for symptomatic MMD. Newly found transfer RNA-derived small RNAs (tsRNAs) perform potential regulatory functions in neovascularization, which is a well-known pathological manifestation of MMD. In this study, the neutrophilic tsRNA transcriptome in aMMD was profiled using next-generation RNA sequencing in five patients and five matched healthy subjects. A negative binominal generalized log-linear regression was used to identify differentially expressed (DE)-tsRNAs in aMMD. Gene Ontology and functional pathway analyses were used to identify biological pathways involved with the targeted genes of the DE-tsRNAs. Four tsRNAs were selected and validated using quantitative reverse transcription polymerase chain reaction. In total, 186 tsRNAs were DE between the two groups. Pathophysiological events, including immune response, angiogenesis, axon guidance, and metabolism adjustment, were enriched for the DE-tsRNAs. The expression levels of the four DE-tsRNAs were consistent with those in the neutrophilic transcriptome. These aberrantly expressed tsRNAs and their targeted pathophysiological processes provide a basis for potential future interventions for aMMD.

## 1. Introduction

Moyamoya disease (MMD) is a chronic progressive cerebrovascular steno-occlusion disorder that affects the bilateral or unilateral main trunks of the circle of Willis, with concomitant abnormal collateral networks [1]. Epidemiological studies have confirmed that MMD has a higher incidence in East Asian countries, including China, than in other countries, showing wide regional variations [2,3]. The initial clinical manifestations of MMD include transient ischemic attack, ischemic and hemorrhagic stroke, headaches, seizures, and cognitive impairment, although those affected can also be asymptomatic [4].

With the increasing availability of non-invasive diagnostic techniques and their popularization in regular medical checkups, the incidence of asymptomatic MMD (aMMD) has been found to be higher than that previously considered [5]. However, our understanding of its pathophysiological progress remains limited, which is reflected in the lack of interventions available for aMMD [6,7]. Previous studies investigating the genetic background and acquired conditions of symptomatic MMD have substantially contributed to our understanding of the pathogenesis of MMD [8,9]. In this study, by focusing on patients with aMMD, especially those without any vascular risk factors, we hope to provide important insights into the natural course of MMD and help develop prophylactic intervention measures to prevent MMD.

Transfer RNA (tRNA)-derived small RNA (tsRNA) is a newly discovered class of non-coding RNAs, with lengths of 15–45 nt [10,11,12]. They are functional RNA populations, implicated in stress and viral infections, cancer, neurodegeneration, metabolic syndromes, and microbiome dysregulation [13]. Recently, studies have identified the role of tsRNAs in cerebral ischemic injury, inflammation, and neovascularization [14,15,16,17], which conforms with the pathological manifestations of MMD.

In the present study, we focused on whether tsRNAs in neutrophils influence aMMD progression, and whether promising therapeutic targets could be identified during this process. To this end, we sequenced neutrophilic tsRNAs in patients with aMMD and probed differentially expressed (DE)-tsRNAs and their targeted pathophysiological processes.

## 2. Materials and Methods

### 2.1. Patients and Controls

This study complied with the Declaration of Helsinki and was approved by the Research Ethics Committee Review Board of Beijing Xuanwu Hospital, Capital Medical University (Beijing, China). Written informed consent was obtained from all individuals involved in the study of blood samples. Patients diagnosed with aMMD using a high-resolution MRI at Xuanwu Hospital were enrolled in our study. Patients diagnosed with moyamoya syndrome (also named secondary moyamoya disease, quasi-moyamoya disease, akin-moyamoya disease, etc.) were denoted as patients with MMD, either bilateral or unilateral, and patients with relations to other underlying diseases, such as autoimmune diseases, atherosclerosis, thyroid disease, neurofibromatosis type 1, tuberous sclerosis, meningitis, down syndrome, cranial irradiation, von Recklinghausen disease, and sickle cell anemia, were noticed and excluded. Patients with hypertension, heart disease, hyperlipidemia, or diabetes, and those taking certain drugs (including anti-inflammatory drugs, antibiotics, antiplatelet drugs, and antiepileptic drugs, among others) were excluded from the study to prevent potential interference with the study. Overall, three male and two female adult patients with aMMD (age range, 45–58 years) and five age- and sex-matched healthy volunteers (age range, 45–58 years) were included in the tsRNA sequencing (seq) study. Peripheral blood samples were extracted into EDTA vacutainer tubes from patients and controls and then used for the purification and isolation of neutrophils, using density gradient centrifugation.

### 2.2. Library Preparation and tsRNA Sequencing 

The procedure for RNA extraction from neutrophils has been described elsewhere. The quantity and quality of RNA were determined using a spectrophotometer. Subsequently, a NanoDrop^®^ ND-1000 instrument (NanoDrop; Thermo Fisher Scientific, Inc., Wilmington, DE, USA) was used to accurately measure the concentration (abs260) and protein contamination (ratio abs260/abs280) of the total RNA samples. The optical density (OD) A260/A280 and A260/A230 ratios of all the samples were within the normal range. Agarose gel electrophoresis was used to assess the integrity of the total RNA samples. No smearing of the ribosomal RNA band indicated that the RNA samples were not degraded.

For efficient reverse transcription, the total RNA samples were then treated using the rtStar™ tsRNA pretreatment kit (cat. no. AS-FS-005; Arraystar Inc., Rockville, MD, USA), which may cause an unavoidable degradation of total RNA, but it could remove possible RNA modifications that may otherwise interfere with the library construction for tsRNA-seq, without a noticeable effect on tRNA quality [18]. The pretreated samples were then used for tsRNA-seq library preparation, using a commercial kit (NEBNext^®^ Multiplex Small RNA Library Prep Set for Illumina, cat. no. 7580; New England Bio Labs, Inc., Ipswich, MA, USA). Library preparation procedures included 3′-adapter and 5′-adapter ligation, cDNA synthesis, polymerase chain reaction (PCR) amplification, and the screening of 134–160 bp PCR-amplified fragments (corresponding to 14–40 nt small RNAs). The completed libraries were quantified using an Agilent 2100 Bioanalyzer (Agilent Technologies, Inc., Santa Clara, CA, USA). According to the quantification results, the libraries were mixed in equal amounts and used for sequencing on an Illumina NextSeq 500 instrument (Illumina, Inc., San Diego, CA, USA).

#### Sequencing Data Analysis

Fast QC (v.0.11.5) software was used to assess the quality of the sequencing. Sequencing fragments were analyzed using the Solexa pipeline (offline Base Caller software, v.1.8). Trimmed fragments were first compared in the tRNA genome database (http://gtrnadb.ucsc.edu/ (v.Release 17, accessed on 30 April 2021)) with Novo Align software (v.2.07.11) to identify known mature tRNA and tRNA precursors. The unrecognized fragments were further compared with other small RNA databases, including those for mRNA, rRNA, snRNA, snoRNA, miRNA, and piRNA. The abundance of tsRNAs was evaluated using sequencing counts and normalized as counts per million of the total aligned reads (CPM). The tsRNAs were filtered if the CPM was < 20 in all the samples. The fold change was then calculated between the aMMD group and the control group to form an expression profile.

DE-tsRNA analysis was performed using the R package edgeR. A tsRNA with a two-tailed *p* < 0.05, after false discovery rate (FDR) correction, according to Benjamini and Hochberg, and a fold change ≥ 2, was defined as DE-one between the two groups. Volcano plots and hierarchical clustering plots were performed in R or Perl for the statistical computing and graphics of DE-tsRNAs.

### 2.3. Bioinformatics Analysis

tsRNAs can interact with Argonaute (AGO) proteins and target RNAs in a manner similar to miRNAs, with seed sequences that have matching profiles similar to those of miRNAs [19]. Based on the presence of binding sites in the 3′-untranslated region, we adopted miRNA target-predicting algorithms, including miRanda (http://www.microrna.org/microrna/home.do (v.3.3a, accessed on 30 April 2021)) and TargetScan (http://www.targetscan.org/vert_71/ (v.7.2, accessed on 30 April 2021)), to identify the potential target genes of the DE-tsRNAs. Subsequently, the predicted target genes were input into the Database for Annotation, Visualization, and Integrated Discovery (DAVID) (http://david.abcc.ncifcrf.gov/ (v.6.8, accessed on 30 April 2021)) with Gene Ontology (GO) (http://www.geneontology.org/ (v.3.5, accessed on 30 April 2021)) to analyze their molecular functions. The Kyoto Encyclopedia of Genes and Genomes (KEGG) (http://www.genome.ad.jp/kegg/ (v.89.1, accessed on 30 April 2021)) database was used for signaling pathway enrichment analysis of the predicted target genes. Similar to GO analysis, statistical algorithms were used in KEGG analysis to analyze the association between a group of target genes and specific signaling pathway entries, and the *p*-value was used to indicate the significance of the association.

### 2.4. Validation by Quantitative Reverse Transcription PCR (qRT-PCR)

The expression of the four tsRNAs selected through bioinformatics analysis was validated using a qRT-PCR assay. In brief, RNA was treated using a rtStar™ tsRNA pretreatment kit (cat. no. AS-FS-005; Arraystar Inc., Rockville, MD, USA) and then converted to cDNA using a rtStar™ First-Strand cDNA Synthesis kit (3′- and 5′-adapters) (cat. no. AS-FS-003; Arraystar Inc., Rockville, MD, USA), according to the manufacturer’s instructions. We performed qRT-PCR in the ViiA 7 Real-time PCR System (Applied Biosystems) using a 2× PCR master mix (AS-MR-006-5; Arraystar Inc., Rockville, MD, USA). The relative tsRNA expression levels were calculated using the relative standard curve method and normalized to U6 levels. All reactions were performed in triplicate. The primers for the tested tsRNAs are listed in Table 1.

## 3. Results

The characteristics of the whole neutrophilic tsRNAs transcriptome of aMMD and controls are provided in Data Supplement I. The heatmap of the correlation coefficient and the hierarchical heatmap from all samples (Data Supplement II) confirmed the reliability and appropriateness of the sample selection, and explicitly showed the extent of intra-case and intra-control variability in both groups.

### 3.1. Differential Expression Analysis

**Figure 1:**
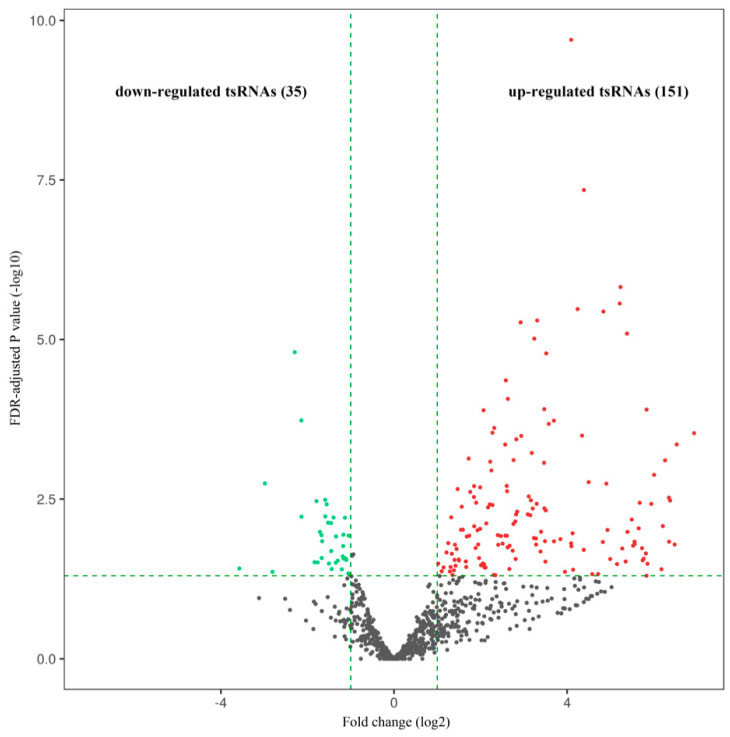
Volcano plot. Red dots represent significantly (fold change ≥ 2.0, false discovery rate (FDR)-adjusted *p* < 0.05) upregulated tsRNAs in aMMD and green dots represent significantly downregulated tsRNAs compared with the control samples. Black indicates tsRNAs that were not differentially expressed.

From the comparative tsRNA sequencing analysis of the neutrophil samples from the patients with aMMD and healthy subjects, 186 tsRNAs were DE between the two groups (fold change ≥ 2.0 and FDR-adjusted *p* < 0.05). Of these, 151 were upregulated and 35 were downregulated (Figure 1). Notably, the top 10 tsRNAs were significantly DE by at least 60-fold between the two groups (FC ≥ 60; FDR-adjusted *p* < 0.05); all of these tsRNAs were upregulated (Table 2). Among the DE-tRNAs, tRF-1 derived from tRNA^Glu^(TTC) and tRF-5b from tRNA^Leu^(TAA) were the most upregulated, whereas tRF-2 from tRNA^Gly^(GCC) was the most downregulated.

### 3.2. Functional Pathway Analysis

**Figure 2:**
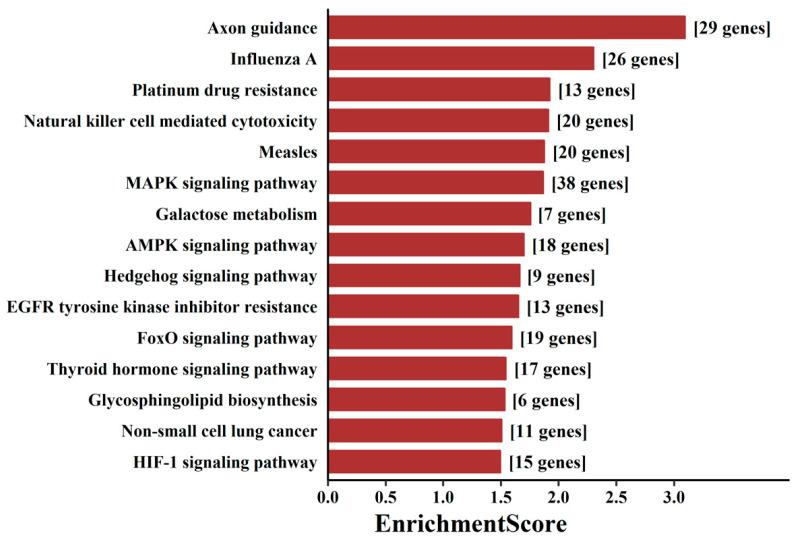
Pathway analysis of targeted mRNAs regulated by DE-tsRNAs. The bar plot showed the top 15 pathways with the highest enrichment score (−log_10_(*p*-value)) and the number of involved genes.

To probe the biological functions of these aberrantly expressed tsRNAs, we used miRanda and TargetScan to predict the putative target genes of the top 20 tsRNAs (ranked by FDR-adjusted *p*-value). The predicted targets of DE-tsRNAs in aMMD were mainly distributed in the nucleus, and their molecular functions were correspondingly implicated in DNA binding transcription factor activity, and the top enriched GO biological processes mainly included the regulation of cellular metabolic processes (Data Supplement III). KEGG pathway analysis revealed 23 significantly enriched pathways (*p* < 0.05) corresponding to the target genes (Data Supplement IV). These pathways were mainly involved in immune response, angiogenesis, metabolism adjustment and axon outgrowth-related pathways, including “axon guidance”, “natural killer cell mediated cytotoxicity”, “MAPK signaling pathway”, “Hedgehog signaling pathway”, “AMPK signaling pathway” and “HIF-1 signaling pathway” (Figure 2).

### 3.3. qRT-PCR Validation 

**Figure 3:**
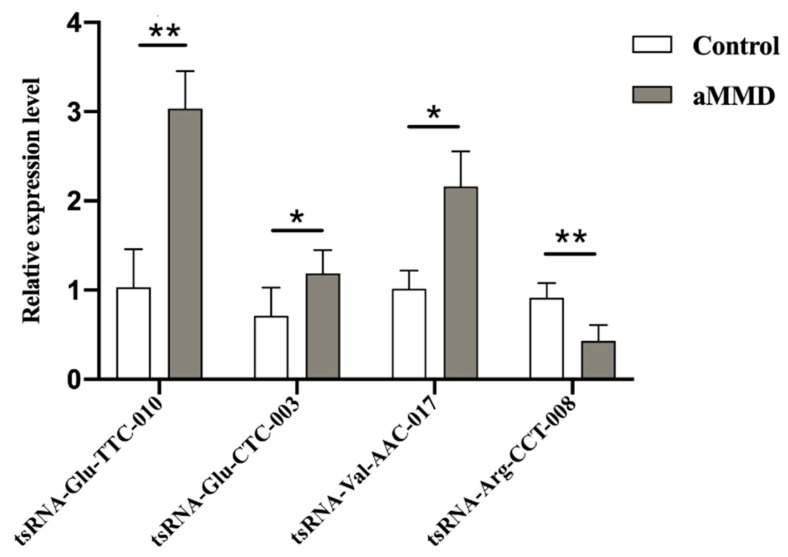
Validation of DE-tsRNAs in aMMD and controls. The comparative bars represented the relative expression level of tsRNA-Glu-TTC-010, tsRNA-Glu-CTC-003, tsRNA-Val-AAC-017, and tsRNA-Arg-CCT-008 in control and aMMD detected by qRT-PCR. *, *p* < 0.05; **, *p* < 0.01.

To further validate the tsRNAs-Seq results, we detected the expression levels of the four candidate DE-tsRNAs, using qRT-PCR in samples from five aMMD patients and five healthy volunteers. tsRNA-Glu-CTC-003, tsRNA-Glu-TTC-010, and tsRNA-Val-AAC-017 were all upregulated, and tsRNA-Arg-CCT-008 was downregulated, showing a similar expression trend as their counterparts in the tsRNAs transcriptome (Figure 3). Additionally, lists of the mRNA targets of the four validated DE-tsRNAs were shown in Data Supplement V.

## 4. Discussion

**Figure 4:**
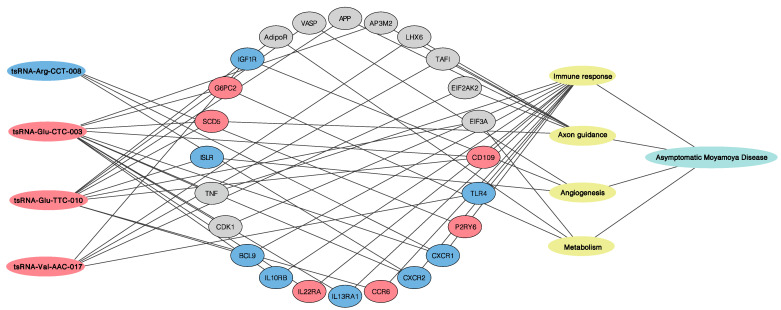
Regulatory networks of the four validated DE-tsRNAs, mRNA targets and pathophysiological processes involved in aMMD. The oblong on the left represented the four RT-PCR validated DE-tsRNAs, the oval in the middle represented the mRNA targets, the oblong on the right represented the four main pathophysiological processes involved in aMMD, and the line represented the predictive connection. The blue indicated the downregulated DE-tsRNA in aMMD (tsRNA-Arg-CCT-008) or the red indicated the upregulated DE-ones in aMMD (tsRNA-Glu-CTC-003, tsRNA-Glu-TTC-010, and tsRNA-Val-AAC-017); similarly, the blue indicted the downregulated mRNA targets, the red indicted upregulated ones, and the grey indicated the mRNA targets regulated by the DE-tsRNA but not differentially expressed in mRNA sequencing data (Data Supplement VI).

This study first profiled the transcriptome of neutrophilic tsRNAs in aMMD. Compared with neutrophils in controls, we identified 109 tsRNAs specifically expressed in the aMMD group. This suggested that aMMD had an unstable status, and the altered expression profile of tsRNAs in neutrophils may partly contribute to disease progression. More importantly, functional pathway analysis showed that the targeted genes of DE-tsRNAs were involved in immune response and angiogenesis, which coincided with the previously accepted pathological manifestation of MMD. In addition, axon guidance and metabolism adjustment, mediated by the HIF-1 signaling pathway, as well as other cascades, also played critical roles, which may explain the specificity of the recruited aMMD patients. In order to strengthen the results deduced from the analyses from the DE-tsRNAs, here we compared the functional pathway analysis of the targeted genes of DE-tsRNAs with the mRNA sequencing data (Data Supplement VI) obtained from the same sample of aMMD and control subjects [20], and we found that consistent with the results from the prediction of putative targeted mRNA (Data Supplement V), the main pathophysiological processes were highlighted in the in-depth analyses result of mRNA sequencing data as well, which supported the roles of the four main pathophysiologic processes in aMMD. As to the specific mRNA targets of DE-tsRNAs, we also compared the mRNA targets of the four tsRNAs validated by qRT-PCR, with the DE-mRNA, detected by the mRNA sequencing to further reinforce the results (Figure 4).

### 4.1. Immune Response

Among the pathophysiologic pathways enriched by DE-tsRNAs, immune cell activation and the release of inflammatory factors were the most widely regulated, highlighting the pivotal role of the immune response in MMD. In line with the results of surgical specimens showing some infiltration of macrophages and T cells in the thickened intima of MMD [21], here we found evidence for possible monocyte, macrophage, and T cell involvement. Key genes such as *HLA-G*, *HLA-DPA1* and *HLA-DQA1* (MHC family), and *CD28* (costimulatory molecule on T cell), *IL-2R*, *CD209* (M2-like markers), *CD109*, *TLR2/4*, and *P2RY6* were all predicted to be regulated by DE-tsRNAs in aMMD, among which, *CD109* was the common target of tsRNA-Glu-TTC-010 and tsRNA-Glu-CTC-003 and upregulated in the neutrophilic mRNA sequencing data; *P2RY6* was the target of tsRNA-Arg-CCT-008 and upregulated in the mRNA sequencing result; and *TLR4* was the target of tsRNA-Val-AAC-017 and downregulated in the mRNA sequencing result (Figure 4 and Data Supplement IV–VI). Moreover, the differentially altered targeted mRNA *FAS* and *FAIMS* were indicative of the possible role of natural killer cells in aMMD. Consistent with previous studies that showed elevated serum and CSF levels of endothelial adhesion molecules in MMD [22], in the present study, *ICAM1/2, CXCR1/2*, and *CCR6* were predicted and regulated, and there was also one pathway highlighted as “cell adhesion molecules” in the in-depth analyses of the DE-mRNA, implicating the transmigration process of leucocytes in aMMD. In particular, *CXCR1/2* were detected as differentially expressed mRNAs from the mRNA sequencing data, and the common targets of tsRNA-Glu-CTC-003 and tsRNA-Arg-CCT-008, and *CCR6* was also targeted by tsRNA-Glu-TTC-010 and upregulated in the mRNA sequencing data (Figure 4 and Data Supplement V–VI). The implication of a possible complex interplay between these immune cells via some targeted genes, such as *IL10RB*, *IL13RA1* and *IL22RA*, predicted to be targeted by tsRNA-Glu-CTC-003 and upregulated in the mRNA sequencing data, and the *BCL9* target of tsRNA-Glu-TTC-010, which was downregulated in the mRNA sequencing data (Figure 4 and Data Supplement V–VI), provides a basis for further exploration in MMD [23]. Meanwhile, immunosuppression may occur, along with immune activation in aMMD, manifesting in processes that inhibit T cell proliferation and Th1/2 differentiation, mediated by cell cycle retardation in the KEGG analyses of the mRNA targets of DE-tsRNA; the negative regulation of T cell activation, enriched by DE-mRNAs, involved genes such as *cyclins*, predicted to be targeted by tsRNA-Val-AAC-017 and differentially expressed in the mRNA sequencing data. Moreover, the FOXO signaling pathway, shown both in the analyses of mRNA targets of DE-tsRNA and DE-tsRNA (Figure 4 and Data Supplement IV–VI), may also contribute to immunosuppression by mediating cellular apoptosis in aMMD [24]. It is worth noting that, in our data, thyroid hormone signaling was enriched for targeted genes of DE-tsRNAs, which supports the theory that increased thyroid function or underlying thyroid autoimmunity is associated with MMD [25].

### 4.2. Cell Proliferation and Axon Guidance

Abnormal cerebral vessel function causes massive neuronal apoptosis, while uncompromised brain tissue invokes many compensatory mechanisms, such as cell proliferation, axon guidance, and angiogenesis [26]. Here, we identified enriched terms implicated in EGFR tyrosine kinase inhibitor resistance and the hedgehog signaling pathway, highlighted both in the GO and KEGG pathway analyses of mRNA targets of DE-tsRNAs and DE-mRNAs, which are critical cascades responsible for cell proliferation, growth, survival, and angiogenesis, in line with the previous discovery in surgical specimens that smooth muscle cells proliferate in the occlusive cerebrovascular lesions in MMD [21]. Moreover, our data implicated DE-tsRNAs target genes in axon guidance with the highest enrichment score (Figure 2) and it was also underlined in the GO analyses of the DE-mRNA as the term “response to axon injury”, where the targeted genes included *TAFI*, *EIF2AK2*, *NLGN3*, *LHX6*, *SCD5*, *ISLR2*, and *AP3M2* (Figure 4 and Data Supplement IV). These genes play critical roles during axon outgrowth, axon repulsion, and axon attraction, and may regulate the regenerative and reparative capacity of neurons during the progression of abnormal vascular networks in MMD. This was also consistent with the results of neuroimaging studies, which showed that chronic ischemia in aMMD decreased the axon density in the white matter and the dendrite density in the cortex [27]. It is worth noticing that *SCD5* and *ISLR* were the predicted targets of tsRNA-Glu-TTC-010 and tsRNA-Glu-CTC-003, and were aberrantly expressed in the mRNA sequencing data (Figure 4).

### 4.3. Angiogenesis and Metabolism Adjustment

In addition, to deal with energy shortages, brain tissues prioritize the supply of energy to the most needed locations to maintain homeostasis, defending against the onset of ischemic or hemorrhagic stroke. This can be deduced from GO terms such as “cellular response to environment stimulus” in the mRNA sequencing and “regulation of cellular metabolic process” in the tsRNA sequencing. Notably, HIF-1 signaling was shown to be activated by DE-tsRNAs (Figure 2), which acted to sense oxygen shortages and redistribute oxygen supply in patients with aMMD. This is particularly important for abnormal blood vessels supplying oxygen and energy to the brain tissues involved [28]. Surgical revascularization can only relieve ischemic symptoms in a minority of patients with MMD, while the activation of the HIF-1 pathway may exert compensatory adjustment to protect MMD patients from symptom attack. However, potential hemorrhagic symptoms, induced by the activation of genes regulating vascular tone and angiogenesis in HIF-1 pathways, should also be considered. In addition, our data indicate that some targeted genes are involved in galactose metabolism. *G6Pase*, *AdipoR*, *IGF1R*, *INSR*, and *INSRR* were predicted to be targeted by DE-tsRNAs (Data Supplement IV); particularly, tsRNA-Glu-CTC-003 targeted *G6PC2* and tsRNA-Val-AAC-017 targeted *IGF1R* were both differentially altered in the mRNA sequencing results (Figure 4 and Data Supplement V and VI), which jointly pointed to the conclusion drawn from a previous study, in which an elevated supply of energy protected patients with MMD from brain dysfunction [29].

There are several limitations to this study. First, despite further analysis of DE-tsRNAs in our sequencing results, validation in larger samples was not conducted due to the difficulty of recruiting more asymptomatic patients. Relatively small sample size and different samples from different subjects might also be partially responsible for the fact that the rank of FC value for the four candidate DE-tsRNAs by the qRT-PCR validation did not match their counterparts in the tsRNAs transcriptome. Second, there was no symptomatic MMD group as a reference. However, we plan to perform a follow-up of these asymptomatic patients and the alterations in their tsRNAs, the possible clinical outcomes of which may help us to better understand the natural course of MMD and its pathological mechanism. Finally, we were unable to conduct further research into the DE-tsRNAs due to a lack of matched animal models and cell lines for MMD.

## 5. Conclusions

This study conducted neutrophilic tsRNA profiling in patients with aMMD and healthy subjects. Pathophysiological processes, including immune response, axon guidance, angiogenesis, and metabolism adjustment, were highlighted by DE-tsRNAs and DE-mRNA in aMMD patients, which supports the potential receptivity of aMMD to medical therapeutics, such as immune-modifying drugs. Further studies will be needed to elucidate the complex mechanism of these tsRNAs and their associated pathways, in order to better understand the pathogenesis of MMD.

## Figures and Tables

**Table 1:** Sequence of primers used for qRT-PCR.

Gene Name	Primer Sequence	Tm (°C)	Product Length (bp)
U6	F: 5′GCTTCGGCAGCACATATACTAAAAT3′ R: 5′CGCTTCACGAATTTGCGTGTCAT3′	60	89
tsRNA-Glu-TTC-010	F: 5′AGTCCGACGATCTCCCACAT3′ R: 5′TCCGATCTAGGAATCCTAACCG3′	60	49
tsRNA-Glu-CTC-003	F: 5′TCTACAGTCCGACGATCTCCCT3′ R: 5′GCTCTTCCGATCTACTAGACCACC 3′	60	46
tsRNA-Val-AAC-017	F: 5′GACGATCGTTTCCGTAGTGTAGTG3′ R: 5′TGCTCTTCCGATCTAAACGTGA3′	60	50
tsRNA-Arg-CCT-008	F: 5′CTACAGTCCGACGATCGCCT3′ R: 5′TGTGCTCTTCCGATCTCAAAAGT3′	60	46

**Table 2:** Top 10 DE-tsRNAs with the highest fold change.

TsRNA_ID	Type	Length	FC (abs)	FDR-Adjusted *p*-Value	Regulation	TsRNA Sequence
tsRNA-Glu-TTC-003	tRF-1	22	122.3993273	0.00029169	up	ATGATGTATGCTTTGTTTCTGTT
tsRNA-Leu-TAA-037	tRF-5b	22	92.58353218	0.00043738	up	GTTAAGATGGCAGAGCCCGGTA
tsRNA-Gly-GCC-022	tRF-1	16	89.6665225	0.01625079	up	GCACGCCCTCCCATTT
tsRNA-Gly-TCC-035	tRF-1	15	83.29000089	0.00332883	up	GCGGGCGGACCTTTT
tsRNA-Leu-TAA-012	tRF-1	16	81.97503369	0.01469735	up	AAGAGGAGTTGTTTTT
tsRNA-Gln-TTG-047	tRF-1	18	81.75096387	0.00300796	up	TTCAAAGGTGAACGTTTT
tsRNA-Val-CAC-007	tRF-5c	28	76.86797098	0.00077968	up	GCTTCTGTAGTGTAGTGGTTATCACGTT
tsRNA-Gly-CCC-050	tRF-1	31	74.16577619	0.00836043	up	AAAGGGTCTTTTTCACCCCGCTGTTGCTCTT
tsRNA-Ala-TGC-001	tRF-1	14	72.54554601	0.03955729	up	AACGGTGACTTTTT
tsRNA-Glu-TTC-003	tRF-5b	22	64.48624603	0.00029169	up	AGTAAGGTCAGCTAAATAAGCT

FC: fold change; FDR: false discovery rate.

## Data Availability

The data presented in this study are available in [Online Data Supplement I–VI].

## Supplementary Materials

The following are available online at https://www.mdpi.com/article/10.3390/cells10051086/s1, Data Supplement I:The characteristics of the whole neutrophilic tsRNAs transcriptome of aMMD and controls, Data Supplement II: The heatmap of the correlation coefficient and the hierarchical heatmap of all involved samples, Data Supplement III: GO analyses results of top twenty DE-tsRNAs, Data Supplement IV: KEGG analyses results of top twenty DE-tsRNAs, Data Supplement V: Lists of mRNA targets of PCR validated DE-tsRNA, Data Supplement VI: The characteristics of the neutrophilic DE-mRNA transcriptome of aMMD and controls. Online Data Supplement I–VI.
