# Supplementary figures and images for: Pathophysiological Significance of Neutrophilic Transfer RNA-Derived Small RNAs in Asymptomatic Moyamoya Disease
Source: Cells. 2021 May 1;10(5):1086. doi: 10.3390/cells10051086 (PMC8147334; doi:10.3390/cells10051086)

A

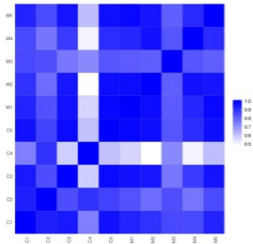

B

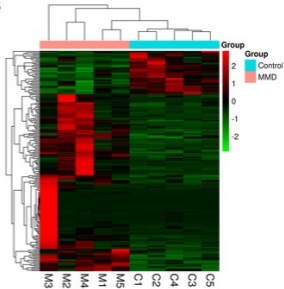

Supplement: Supplementary file 1 [file cells-10-01086-s001.zip › cells-1149161 3rd supplementary materials/Data Supplements/Data Supplement II.pdf]
